# A retrospective study of outcomes of device-associated osteomyelitis treated with daptomycin

**DOI:** 10.1186/s12879-016-1590-3

**Published:** 2016-06-24

**Authors:** Elizabeth D. Hermsen, Luke Mendez-Vigo, Elie F. Berbari, Thomas Chung, Minjung Yoon, Kenneth C. Lamp

**Affiliations:** Merck & Co., Inc., 2000 Galloping Hill Road, Kenilworth, NJ 07033 USA; Department of Pharmacy Practice, College of Pharmacy, University of Nebraska Medical Center, Omaha, NE USA; Department of Medicine, Division of Infectious Diseases, Mayo Clinic College of Medicine, 200 1st St, SW, Rochester, MN 55905 USA

**Keywords:** Daptomycin, Device-associated osteomyelitis, Safety

## Abstract

**Background:**

Daptomycin appears well tolerated and effective for osteomyelitis treatment. However, limited data exist regarding daptomycin use for treatment of device-associated osteomyelitis (DAO).

**Methods:**

We used a retrospective, observational database (Cubicin® Outcomes Registry and Experience [CORE® 2007–2009]) that assessed patients treated with daptomycin to evaluate the characteristics of patients with DAO, outcomes after daptomycin treatment, and safety of daptomycin in this setting. Information from 54 institutions for patients with prosthetic joint infection (PJI) and other hardware-associated osteomyelitis (OHAO) who received daptomycin from January 2007 to December 2008 with follow-up data in 2009 was collected using a standardized data collection form.

**Results:**

Eighty-two patients receiving daptomycin were identified in CORE 2007–2009; 48 patients (59 %) had follow-up data. Sixty-seven percent of patients had received a previous antibiotic. Surgical intervention was similar between the 2 groups: PJI, 22 of 27 (82 %) and OHAO, 17 of 21 (81 %). However, device removal or replacement was more frequent in the PJI patients (17 of 27, 63 %) than in the OHAO patients (8 of 21, 38 %). Clinical success was reported in 22 of 27 (82 %; 95 % confidence interval [CI], 62–94 %) patients with PJI and 18 of 21 (86 %; 95 % CI, 64–97 %) patients with OHAO at follow-up (13–402 days). Adverse events occurred in 8 of 50 (16 %) patients in the safety population and did not differ by daptomycin dose.

**Conclusion:**

Daptomycin appeared effective and well tolerated in patients with DAO, including PJI or OHAO.

## Background

Daptomycin is a cyclic lipopeptide with potent activity against a broad range of Gram-positive organisms [1–3]. Daptomycin is approved for the treatment of complicated skin and skin structure infections due to certain Gram-positive bacteria and for the treatment of *Staphylococcus aureus* bacteraemia and right-sided infective endocarditis [4].

Device-associated osteomyelitis (DAO), such as prosthetic joint infection (PJI), is an increasing and costly problem associated with high morbidity rate and prolonged hospitalization [5]. Rates of PJI have been reported to be 0.5–1.0 % after hip replacement and 1–2 % after knee replacement surgery [6]; these procedures are performed annually in 12.0 and 27.9 per 10,000 adults age 45–64 years, respectively, and 33.0 and 83.4 per 10,000 adults age 65 years and older, respectively (based on 2007 U.S. hospital discharges) [7]. While many organisms can cause PJI, methicillin-susceptible *S. aureus* (MSSA), methicillin-resistant *S. aureus* (MRSA), and coagulase-negative staphylococci (CoNS) are the most frequently reported [8, 9].

Current data suggest that daptomycin appears to be an effective and well-tolerated treatment option in patients with osteomyelitis [10–13]. However, limited data are available describing the clinical experience of daptomycin specific to DAO [5, 8, 13, 14].

The purpose of this study was to describe the characteristics and management of patients with DAO treated with daptomycin, including those with PJI and other hardware-associated osteomyelitis (OHAO), and to assess clinical outcomes and safety using retrospective data collected in an observational database.

## Methods

This study was performed at multiple institutions of investigators involved in the Cubicin® Outcomes Registry and Experience (CORE® 2007–2009) program. CORE was a retrospective observational database designed to assess the demographic characteristics and clinical outcome of patients treated with daptomycin. After institutional review board (IRB) approval from the Chesapeake IRB (CRRI 0504020) with a waiver of informed consent and at each participating site requiring local approval, 54 institutions in the United States collected information using a standardized data collection form from January 2007 to December 2008. Data from patients treated with daptomycin included age; sex; weight; renal function; type of dialysis (if applicable); comorbidities; daptomycin initial and final dose, dosing interval, and length of therapy; prior, concomitant, and follow-up antibiotic therapy; infection details; outcomes; discharge information; and safety assessments. Detailed CORE methodology has been previously published by Rolston *et al* [15].

### Patients

The study population included patients from CORE with DAO (PJI and OHAO). Patients were included if they were diagnosed with osteomyelitis in the presence of prosthetic device/hardware, completed therapy with daptomycin, and had a documented follow-up assessment in 2009. A total of 82 patients were identified as having DAO and successfully completing daptomycin therapy in CORE 2007–2008.

### Clinical outcome

Patient clinical outcome was categorized as cure, improved, failure, or non-evaluable. Clinical cure was defined as at least 1 of the following: clinical signs and symptoms resolved; no additional antibiotic therapy necessary; and/or negative culture reported at the end of therapy. An improved clinical outcome was defined as partial resolution of clinical signs and symptoms and/or additional antibiotic therapy warranted at the end of therapy. Clinical failure was defined as any of the following: inadequate response to therapy (worsening or new/recurrent signs and symptoms), a required change in antibiotic therapy, or positive culture reported at the end of therapy. Clinical outcome was considered non-evaluable if the information was insufficient to determine response. Clinical success was defined as the sum of patients with clinical cure and those who had improved clinical outcome. Outcome was determined at the last follow-up assessment after daptomycin therapy was completed.

### Data analysis

Data analysis was conducted using SAS 9.1 (SAS Institute, Inc., Cary, North Carolina). The efficacy analysis included patients with evaluable follow-up data in CORE 2009. The safety analysis included all treated patients with DAO who completed therapy with daptomycin and had a documented follow-up assessment in CORE 2009, including those non-evaluable for clinical outcome. The Fisher’s exact test and a Kaplan-Meier analysis were used to determine statistical significance, which was defined as a *p* value < 0.05; an exact 95 % confidence interval (CI) was also calculated.

## Results

### Patient demographics and characteristics

Of the 82 identified patients with DAO who successfully completed therapy with daptomycin, 32 (39 %) were from institutions that did not participate in CORE 2009 and had no follow-up information, while 48 (59 %) had follow-up information in CORE 2009 and were evaluable. Two additional patients were non-evaluable and were only included in the safety analysis. In the evaluable population, PJI was diagnosed in 56 % (27 of 48) and OHAO in 44 % (21 of 48) (Table 1). The most common pathogen identified was MRSA in 27 % (13 of 48) of patients (Table 2).

**Table 1:** Demographic characteristics of patients with device-associated osteomyelitis treated with daptomycin in the CORE database

Characteristic	No follow-up in CORE 2009	Evaluable population	
		PJI	OHAO
	(*n* = 32)	(*n* = 27)^a^	(*n* = 21)^a^
Sex, n (%)			
Female	18 (56.3)	12 (44.4)	12 (57.1)
Male	14 (43.8)	15 (55.6)	9 (42.9)
Median weight, kg (range)	81.4 (37.1–120.0)	86.4 (44.1–128.9)	79.0 (40.0–136.4)
Age group (y), n (%)			
≤ 50	6 (18.8)	4 (14.8)	13 (61.9)
51–65	14 (43.8)	12 (44.4)	7 (33.3)
≥ 66	12 (37.5)	11 (40.7)	1 (4.8)
In community 48 h prior to receiving daptomycin, n (%)	15 (46.9)	13 (48.1)	11 (52.4)
ICU stay during daptomycin, n (%)	7 (21.9)	1 (3.7)	0 (0.0)
Initial creatinine clearance	5 (15.6)	2 (7.4)	0 (0.0)
< 30 mL/min, n (%)			
Surgical intervention, n (%)	22 (68.8)	22 (81.5)	17 (81.0)
At least 1 pathogen identified, n (%)	22 (68.8)	20 (74.1)	18 (85.7)
Median initial daptomycin dose, mg/kg (range)	6 (4–13.5)	6 (4–9)	6 (4–8)
Median duration of daptomycin, days (range)	35 (2–78)	41 (6–70)	40 (8–62)

*CORE* Cubicin® Outcomes Registry and Experience, *ICU* intensive care unit, *OHAO* other hardware-associated osteomyelitis, *PJI* prosthetic joint infection

^a^One patient in each of the PJI and OHAO groups was not included in the evaluable population

**Table 2:** Microbiology of patients with device-associated osteomyelitis treated with daptomycin in the CORE database

Pathogens, n (%)^a^	No follow-up in CORE 2009	Evaluable population	
		PJI	OHAO
	(*n* = 32)	(*n* = 27)	(*n* = 21)
*Staphylococcus aureus* (MRSA)	9 (28.1)	6 (22.2)	7 (33.3)
*Staphylococcus aureus* (MSSA)	2 (6.3)	3 (11.1)	4 (19.0)
Other *Staphylococcus aureus* ^b^	1 (3.1)	1 (3.7)	0 (0.0)
Coagulase-negative staphylococci	3 (9.4)	6 (22.2)	3 (14.3)
*Enterococcus* spp.	4 (12.5)	3 (11.1)	3 (14.3)
Vancomycin-resistant enterococci	1 (3.1)	1 (3.7)	2 (9.5)
*Streptococcus* spp.	0 (0.0)	0 (0.0)	1 (4.8)
Other	7 (21.9)	3 (11.1)	1 (4.8)

*CORE* Cubicin® Outcomes Registry and Experience, *MRSA* methicillin-resistant *S. aureus*, *MSSA* methicillin-susceptible *S. aureus*, *OHAO* other hardware-associated osteomyelitis, *PJI* prosthetic joint infection

^a^Patients may have had more than one pathogen

^b^Other *S. aureus* includes methicillin susceptibility unknown

### Daptomycin dosage regimen

The median (range) initial daptomycin dose in all evaluable patients was 6 (4.0–9.0) mg/kg (Table 1). The median (range) duration of daptomycin therapy was 41 (6–70) days (Table 1). A dose of ≥ 6 mg/kg was received by 22 of 27 (81 %) and 16 of 21 (76 %) evaluable patients with PJI and OHAO, respectively. Six patients (4 [15 %] PJI; 2 [10 %] OHAO) received a daptomycin dose of ≥ 8 mg/kg.

#### Prior antibiotic therapy

Previous antibiotic use, including multiple antibiotic regimens, occurred in most patients (PJI, 18 of 27 [67 %]; OHAO, 14 of 21 [67 %]). Vancomycin, the most common prior antibiotic, was administered in 37 % (10 of 27) and 43 % (9 of 21) of PJI and OHAO patients, respectively. The median (range) duration of previous antibiotic therapy was 10 (1–90) days in patients with PJI and 10 (2–26) days in patients with OHAO. The most common reason for switching to daptomycin among evaluable patients was a narrowed spectrum in 8 of 18 (44 %) PJI and 7 of 14 (50 %) OHAO patients.

#### Surgery

The percentage of patients who received surgical intervention was similar between the two groups: approximately 82 % for PJI, and 81 % for OHAO (Table 1). However, device removal or replacement was more frequent in the PJI patients (17 of 27 [63 %]) than in the OHAO patients (8 of 21 [38 %]).

#### Outcomes

Clinical success was reported in 22 of 27 (82 %; 95 % CI, 62–94 %) patients with PJI and 18 of 21 (86 %; 95 % CI, 64–97 %) patients with OHAO at follow-up, ranging from 13 to 402 days after therapy. Failures were reported in two patients within 30 days, one within 30 to 60 days, two within 61 to 180 days, and three after more than 180 days of follow-up. Kaplan-Meier analyses stratified by dose of < 6 mg/kg, 6 to < 8 mg/kg, and ≥ 8 mg/kg showed an overall difference (*p* = 0.001) in the median time to failure (not shown). Although the longest time to failure was observed in those receiving daptomycin 6 to <8 mg/kg, the clinical significance should be interpreted cautiously due to small sample sizes for the < 6 mg/kg (*n* = 10) and ≥ 8 mg/kg (*n* = 6) groups. Kaplan-Meier analysis did not show a difference in outcomes by device type. Success was reported in 77 % (10 of 13) of patients with MRSA, 43 % (3 of 7) of those with MSSA, and 100 % (9 of 9) of those with CoNS. Surgical removal or replacement for either device tended to show better success rates; PJI: 8 of 8 (100 %) vs. 10 of 13 (77 %), *p* = 0.3; and OHAO: 15 of 17 (88 %) vs. 7 of 10 (70 %), *p* = 0.3.

### Safety

No deaths or serious adverse events (AEs) were reported. Twelve AEs occurred in 8 of 50 (16 %) patients in the safety population and did not differ by daptomycin dose; all AEs were mild. The most common AE was increased creatine phosphokinase (CPK) in 6 patients. Ten AEs were considered possibly treatment related including all CPK AEs. Daptomycin therapy was discontinued in three patients because of AEs (increased CPK, increased CPK with myalgia, and increased alanine aminotransferase and aspartate aminotransferase). Daptomycin was also temporarily discontinued in one patient because of increased CPK.

## Discussion

Daptomycin is a potent antibiotic with activity against many Gram-positive organisms. Previous studies have demonstrated the efficacy and safety of daptomycin in the treatment of osteomyelitis [10–13, 16]. However, data on the use of daptomycin in DAO are limited [5, 14]. In this report, the clinical success observed in 83 % of all evaluable patients is consistent with those of previously published results. Similar results were reported for osteomyelitis and orthopedic device infections in a European CORE analysis where clinical success at follow-up was 86 % [17]. Patients who received daptomycin at a dose of 6 to < 8 mg/kg had a better outcome than patients receiving doses < 6 mg/kg or ≥ 8 mg/kg, although sample sizes in the latter two groups were small.

The present analysis has several limitations. DAO is relatively rare, with reported rates of 0.5–1.0 % after hip replacement and 1–2 % after knee replacement surgery [6], resulting in a limited pool of potential patients for analysis. Furthermore, because data were collected retrospectively from chart documentation, the number of patients with follow-up data in 2009 was only slightly more than half of those with available records. Follow-up duration was uncontrolled and limited. In addition, data were collected during daptomycin for surgery but were not collected during follow-up. The presence of Gram-negative pathogens might have been under-reported; therefore, the results should be interpreted in the context of these limitations.

Daptomycin is a concentration-dependent antibiotic. Several in-vitro and animal model studies have shown that high dose daptomycin results in enhanced killing and a lower rate of emergence of resistance compared to standard dosing [18]. Clinical studies have followed demonstrating that high-dose daptomycin is generally well-tolerated and results in good clinical response in difficult to treat infections due to *S. aureus* or enterococci [18, 19]. Although the current study had a small number of patients receiving higher doses, more recent studies in bone and joint infections are reporting on the outcomes with high-dose daptomycin [20].

As noted earlier, data on daptomycin in DAO are limited. A previous small, randomized, controlled trial that evaluated the safety and efficacy of daptomycin versus standard-of-care therapy (vancomycin, teicoplanin, or semisynthetic penicillin) in patients undergoing two-stage revision arthroplasty for PJI demonstrated clinical success in 14 of 24 (58.3 %) and 14 of 23 (60.9 %) patients in the daptomycin 6- and 8-mg/kg groups, respectively, compared with 8 of 21 (38.1 %) in the standard-of-care group [8]. A retrospective review of 14 patients treated with daptomycin for hip or knee PJI who were evaluable for efficacy reported an overall success rate of 79 % [9]. Furthermore, a retrospective analysis of osteomyelitis cases in the European CORE database reported success rates with daptomycin treatment in 71 % (41 of 58) of patients who retained a permanent prosthetic device and 80 % (16 of 20) of patients who retained a temporary prosthetic device [13]. A trend toward higher success rates for patients receiving daptomycin who have had their hardware removed has been seen [13, 21]. A recent multicenter observational retrospective study of retained PJI supports this trend. Despite using a high dose of daptomycin, 10 mg/kg/d with rifampin, for acute PJI due to fluoroquinolone-resistant staphylococci, Lara-Tamayo et al. observed a 50 % failure rate (9 of 18 patients) but did not detect an increase in daptomycin minimum inhibitory concentration [14]. In contrast, in this retrospective study, higher rates of clinical success were reported (82 % of patients with PJI and 86 % of patients with OHAO) at follow-up, ranging from 13 to 402 days after therapy.

Previous analyses have reported on the safety of daptomycin in patients with osteomyelitis [10, 13, 22]. In the current retrospective analysis, daptomycin also appeared well tolerated. The most commonly reported AE was increased CPK in 6 of 48 patients. This is consistent with other clinical trials and database analyses of daptomycin at doses ≥ 6 mg/kg in the treatment of osteomyelitis, in which CPK elevations have been reported in 2.5–8.3 % of patients [8, 22].

Guidelines for the diagnosis and management of PJI published in 2013 by the Infectious Diseases Society of America recommend daptomycin at 6 mg/kg as alternative treatment for PJI related to MSSA, MRSA, and *Enterococcus* spp with a level of evidence of BIII (moderate evidence, based on opinions of respected authorities, expert committee reports, clinical experience, or descriptive studies) [23]. The present study supports the recommended daptomycin dose of 6 mg/kg for the treatment of PJI caused by these pathogens; however, the level of evidence emphasizes the importance of additional data.

## Conclusions

Daptomycin appeared effective and well tolerated in the treatment of patients with DAO, including PJI or OHAO. Further studies are warranted to confirm these findings.

## Abbreviations

AE, adverse event; CI, confidence interval; CoNS, coagulase-negative staphylococci; CORE®, Cubicin® Outcomes Registry and Experience; CPK, creatine phosphokinase; DAO, device-associated osteomyelitis; MRSA, methicillin-resistant *Staphylococcus aureus*; MSSA, methicillin-susceptible *Staphylococcus aureus*; OHAO, other hardware-associated osteomyelitis; PJI, prosthetic joint infection
